# Supplementary material for: MetaRibo-Seq measures translation in microbiomes
Source: Nat Commun. 2020 Jun 29;11:3268. doi: 10.1038/s41467-020-17081-z (PMC7324362; doi:10.1038/s41467-020-17081-z)
Supplement: Supplementary file 10 — Supplementary Data 7 [file 41467_2020_17081_MOESM10_ESM.zip › File2/Confidence_High_Taxonomy/443270_out.krona.html]

Javascript must be enabled to view this page.

members
magnitude
magnitudeUnassigned
count
unassigned
taxon
rank

443270\_out

2

2
superkingdom
2

1239
phylum
2

186801
2
class

order
2
186802

186803
family
2

1407607
1
genus

1
species
1150298

SRS097889\_contig\_number\_4453

genus
1
572511

2292975

SRS146813\_contig\_number\_16739
species
1
